# Eeny, meeny, miny, moe—Summer time and out are you? The working population in the EU would likely benefit from elimination of daylight saving time

**DOI:** 10.1007/s00508-023-02311-3

**Published:** 2023-12-08

**Authors:** Eva S. Schernhammer, Susanne Strohmaier, Philip Vonderlind

**Affiliations:** 1 Department of Epidemiology, Center for Public Health, Medical University of Vienna, Kinderspitalgasse 15, 1090 Vienna, Austria; 2 Channing Division of Network Medicine, Department of Medicine, Brigham and Women’s Hospital and Harvard Medical School, 181 Longwood Avenue, 02115 Boston, MA USA; 3 Institute for Informatics, Technical University of Vienna, Favoritenstr. 9–11, 1040 Vienna, Austria

**Keywords:** Day light saving time, European Union, Circadian system, Summer time

## Abstract

Daylight (saving) time (DST) is an over one century old practice to maximize the overlap between natural day light and individual active time (i.e., non-sleep time). Whether to abandon the practice is subject to an ongoing, twice a year intensifying debate. A request to abandon the practice is based on the lack of benefits in terms of energy savings and potential negative health effects. We present a tool that captures one key aspect of importance to the circadian system: maximizing the overlap of natural day light with human active time, focusing on early morning light exposure as the primary stimulus for our circadian system. Based on publicly available data we incorporated an approximation of the 27 European Union (EU) countries’ spatial population distribution into a calculation of average exposure to morning sunlight under DST or no DST conditions for each EU27 country and the entire region. An online app offers visualization of these differences on the country level alongside a population-weighted average for the EU27. Our findings support that the majority of the EU’s working population would likely benefit from the elimination of daylight saving time if maximizing an adequate morning stimulus is the primary goal and adjusting actual time zones or biennially changing the clock is not an option.

Daylight (saving) time (DST) is an over one century old practice to maximize the overlap between natural day light and individual active time (i.e. non-sleep time). It requires advancing clocks during the summer months (for later sunrises yet longer evening daylight). Its introduction was first contemplated in 1895 [1] 20 years after the widespread introduction of artificial/electric light began (Thomas Edison), enabling the second industrial revolution. Daylight saving time was subsequently implemented for the first time in the Austrian-Hungarian empire in 1916 (30 April). Since then, it has intermittently been adopted across the globe, although the energy crisis of the 1970s ushered in one of the longest time periods of constant summer time clock advance (and back in the fall) in large parts of Europe and North America.

With three generations of scientists in the USA and Europe having now grown up with daylight saving time arrangements (meaning, biennially changing lighting conditions during their entire life), and a growing number of studies to assess its impact, a discussion had emerged to abandon the practice, and it appeared near certain in August 2018 that the European Union (EU) would “say goodbye to daylight saving time” for good on 28 October 2018 [2]. The request to abandon the practice was based on the lack of benefits in terms of energy savings as outlined in a recent report [3] and potential negative health effects [4]. A general consensus was reached that ending seasonal time changes needed to be implemented in consensus among member states (to not disrupt EU internal markets), and a survey showed that 84% of responders were in favor of ending the practice of biannual clock changes [5].

The subsequent debate derailed this undertaking as no consensus could be reached about which time to keep permanently—summer or winter time—hence a decision was ultimately postponed. That debate, however, disregarded what used to be normal time before the introduction of daylight saving time (namely winter time), instead considering the full adoption of summer time.

With people increasingly suffering from a growing inability to cope with clock changes [3], aggravated by to too much darkness and too little sunlight in the mornings, i.e., the conditions under which the circadian system is prone to become desynchronized [6], here, in anticipation of future discussions, we present a simple tool to quickly capture a key aspect for the circadian system: maximizing the overlap of natural daylight and human active time, focusing on early morning light exposure as the primary stimulus for our circadian system, a lack of which could lead to a mismatch in the temporal synchronization of the circadian rhythm to working hours [7, 8].

Based on publicly available data we incorporated an approximation of the 27 EU (EU27) countries’ spatial population distribution 2018–2022 (Eurostat Urban Audit [9]) into our calculation of average exposure to morning sunlight under DST or no DST conditions for each EU27 country and the entire region. Specifically, we calculated the difference between 9 am in the respective time zone (a typical work starting time) and the average sunrise time (calculated using the Python package Astral [9]) during the summer period, i.e., last Sunday in March (27th) until last Sunday in October (30th) in 2022 and the winter period (January 1st to March 26th and October 31st to December 31st, 2022) for the three most populated cities in each country with or without consideration of DST. Our online app provides visualization of these differences on the country level alongside a population-weighted average for the EU27.

As can be seen, during the summer period (Fig. 1c, d) the population-weighted difference between sunrise and 9 am averages 3:40 h, and 2:41 h with DST. During the winter period (Fig. 1a, b), the population-weighted average time of morning sun exposure is reduced to 1:27 h and is 0:27 min only with DST. This implies that the amount of time spent in morning daylight on a typical workday is going to be filled with ample sun/light exposure in the summer season, regardless of which condition (± DST) and would arrive at a very low minimum during the winter season particularly under the DST condition. These numbers would be naturally further aggravated if assuming that work starts earlier, e.g., at 8 am, or earlier.

**Fig. 1:**
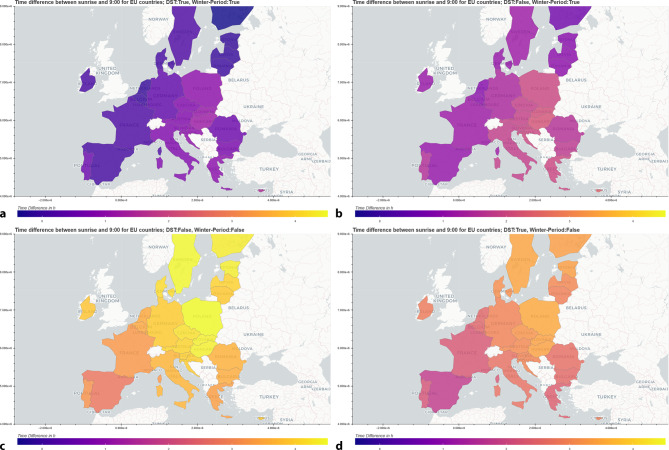
Difference in exposure to morning sunlight with or without daylight saving time (DST). We visualize the difference between average sunrise time and 9 am in the respective time zone. The color shading reflects this time difference, with colors towards the *purple* end indicative of smaller, and colors towards the brighter *yellow* indicative for longer time duration between sunrise (and work start at 9 am) under DST conditions versus not in the summer and winter period. Map of European Union countries, showing population-weighted differences between sunrise and 9am **a** in the winter period with DST, **b** in the winter periods without DST, **c** in the summer period without DST, and **d** in the summer period with DST. App: https://huggingface.co/spaces/philip-vonderlind/Circadian_Rythm

From a public health standpoint, other factors than the spatial distribution of the population also deserve attention, such as the prevalence of different chronotypes (i.e., preferred sleep and wake hours—early, average or late), the distribution of typical work hours (i.e., 0800–1600, or 0900–1700, siesta yes or no) or the prevalence of seasonal affective disorder and general well-being [10].

In summary, the majority of the European Union’s working population would likely benefit from the elimination of daylight saving time if maximizing an adequate morning stimulus is the primary goal and adjusting actual time zones or biennially changing the clock is not an option.
